# Immunity and Treatment of Sporotrichosis

**DOI:** 10.3390/jof4030100

**Published:** 2018-08-20

**Authors:** Laura Cristina García Carnero, Nancy Edith Lozoya Pérez, Sandra Elizabeth González Hernández, José Ascención Martínez Álvarez

**Affiliations:** Departamento de Biología, División de Ciencias Naturales y Exactas, Campus Guanajuato, Universidad de Guanajuato, Noria Alta s/n, col. Noria Alta, C.P., Guanajuato Gto. 36050, Mexico; laura_cgc@hotmail.com (L.C.G.C.); nelppat@hotmail.com (N.E.L.P.); se.gonzalezh@gmail.com (S.E.G.H.)

**Keywords:** immunity, sporotrichosis, host-defense, antifungal

## Abstract

Species of the *Sporothrix* complex are the etiological agents of sporotrichosis, an important subcutaneous mycosis with several clinical forms and an increasing incidence around the world that affects humans and other mammals. The immunological mechanisms involved in the prevention and control of this mycosis are not entirely understood. Many reports have suggested that cell-mediated immunity has an essential role in the development of the disease, being the primary response controlling it, while only recent data supports that the humoral response is essential for the appropriate control. This mycosis is a challenge for diagnosis since the culture and isolation of the organism are time-consuming and complicated; reasons that have led to the study of fungus antigenic molecules capable of generating a detectable humoral response. The treatment for this disease includes the use of several antifungal drugs like itraconazole, amphotericin B, caspofungin, fluconazole, and the combination between them among others such as the extract of *Vismia guianensis*.

## 1. Sporotrichosis

Sporotrichosis is a fungal infection with a worldwide distribution that is prevalent mainly in tropical and subtropical regions and predominates in South Africa, America (Brazil, Peru, Colombia, Guatemala, Mexico, and the United States), Asia (Japan, China, India) and Oceania, but is rarely found in Europe [1,2]. It presents a wide range of clinical manifestations, from localized to disseminated infections including lymphocutaneous, extracutaneous, and disseminated. A total of 95% of cases are cutaneous lymphatic sporotrichosis, spreading through the lymph nodes affecting mainly the face and upper and lower limbs. The cutaneous fixed form (30% of sporotrichosis cases) occurs by ulcerative or verrucous nodules with well-defined borders, most probably due to the good immune response of the host. The cutaneous disseminated form represents only 8% of cases, and is mainly associated with immunosuppressive processes or diseases such as diabetes, lymphomas, HIV-AIDS, treatments with systemic corticosteroids, and chronic alcoholism [3]. Alternative forms of extracutaneous sporotrichosis have increased in recent years and they involve the lungs, the osteoarticular system, and the central nervous system [2,4,5]. Systemic and disseminated infections are present in immunocompromised individuals, affecting several organs [6]. These clinical manifestations depend on different factors such as the immune response of the host, the virulence of the strain, the amount of inoculum, and the depth of inoculation [7]. In humans, like in other mammals, the route of entry is cutaneous by traumatic injury with contaminated material by the fungus or transmission by bites or scratches from infected animals [8]. Recently, a complex of different species that cause sporotrichosis have been proposed based on molecular and morphological analysis [9,10].

## 2. *Sporothrix schenckii* Complex

Species of the *Sporothrix* complex are thermodimorfic fungi, with a saprophytic phase that grows at 25 °C as a mycelium, and a parasitic phase that grows at 35–37 °C as a “cigar-shaped” yeast-like morphology. The *Sporothrix* complex show differences in geographic distribution, frequency, ecological niche, and virulence of the members of this genus. Species of this complex have been reported as agents of sporotrichosis in animals and humans and are known as the clinical clade: *S. schenckii sensu stricto*, *Sporothrix brasiliensis*, *Sporothrix globosa*, and *Sporothrix luriei*, *Sporothrix mexicana*, *Sporothrix pallida* (*Sporothrix albicans*), and *Sporothrix chilensis* are environmental isolates but can behave as opportunistic pathogens in immunosuppressed individuals [9]. *S. schenckii* and *S. globosa* have been isolated from humans, animals, and soils containing cellulose, organic matter, grasses, woods, leaves, and branches, following a route of infection through the contaminated material [9,11,12]. High virulence has been reported in *S. brasiliensis*, a species associated with zoonotic transmission through scratches or bites from animals infected with this fungus [13]. The virulence level in a murine model has shown that *S. brasiliensis* is the most virulent species, followed by *S. schenckii*, and then *S. globosa* [14]. In Brazil, *S. brasiliensis* has a high prevalence in cats, and interestingly, the same genotypes were observed in both human and cats [15].

Several molecular methods for the identification of the different *Sporothrix* species were applied, and these were aimed at different targets such as the protein-coding genes calmodulin (*CAL*), β-tubulin (*Bt2*) and chitin-synthase 1 (*CHS1*), the 18S rDNA, and the internal transcribed spacer (*ITS*) [1,16] (Table 1) [9,17,18,19,20,21,22]. The DNA data from three different loci (*CAL*, *Bt2*, and *CHS*) of *Sporothrix* were analyzed and used to group the isolates into six putative cryptic species with a degree of geographical specificity. These results are supported by the suggestion that different species of *Sporothrix* exist, based on the analysis of the *ITS* region sequences from clinical and environmental samples [19]. Later on, different *Sporothrix* strains were characterized and three new species were differentiated based on the *CAL* sequence, *S. globosa*, *S. brasiliensis*, and *S. mexicana*. The *CAL* sequence has been reported as the best marker and more phylogenetically informative locus [9], and is now considered as a standard for the molecular identification of *Sporothrix* [23].

## 3. Virulence Factors of *Sporothrix* spp.

The virulence factors related to the *Sporothrix schenckii* complex are probably related to the clinical manifestations of the mycosis, but are not well known. Some of the reported molecules and mechanisms that act as virulence factors are glycoproteins, secreted proteins, extracellular vesicles, melanin, ergosterol peroxide, and the dimorphism of the fungus [16,24,25,26,27].

Dimorphism is the ability of some fungi to exhibit a phenotypic duality that leads to a cellular differentiation process, which might be related to pathogenicity. A hybrid dimorphism-regulating histidine kinase that may be involved in the dimorphic transition was reported in *Sporothrix* (SsDRK1). In other dimorphic fungal pathogens, this protein regulates the expression of virulence genes and pathogenicity in vivo [16,26,28]. Thermotolerance is another fundamental virulence factor for the survival of the fungus in the host [29]. Environmental strains of *Sporothrix* cannot adapt to the temperature of the host body, and therefore cannot produce infection [16]. Melanin, also referred to as “fungal armor”, is an important virulence factor not only for *Sporothrix*, but also for many pathogenic fungi. This pigment in the cell increases fungal survival in the host, given that it makes the cell more resistant to phagocytosis and killing by macrophages and neutrophils. Both morphologies of *Sporothrix* are able to produce melanin through the 1,8-dihydroxynaphthalene (DHN) and l-DOPA pathways. In *S. brasiliensis*, *S. schenckii*, and *S. globosa*, a third l-tyrosine (pyomelanin) pathway was observed in the stationary phase of mycelium and yeast [30,31]. This component has been reported to protect from nitrogen derived oxidants and the antifungal Amphotericin B [26,29]. Several vesicle-associated proteins have been reported in *Sporothrix*. Extracellular cell wall glucanase is a protein transported by vesicles that contributes to the fungal virulence by inducing macrophages and other host cells lysis due to the remodeling of their surface [25]. Superoxide dismutase (SOD), another cell wall protein, contributes to the growth and survival of the pathogen under conditions of oxidative stress, just like inside macrophages, and its presence has been reported in *Sporothrix* [26]. Certain proteases such as Proteinase I play an essential role in the interaction of this fungus with the host cells, given that it is associated with the ability of *Sporothrix* to invade the cutaneous tissues [16,32]. Adhesins are an important virulence factor in every pathogenic fungus, in the case of *Sporothrix*, they are in charge of mediating the binding of yeast to the dermal matrix and to fibronectin. The main adhesin reported during sporotrichosis is a glycoprotein of 70 kDa, known as Gp70. In addition, this protein plays a crucial role in modulating the host immune response, is highly immunogenic, and its detected by all sera of infected mice [33].

All of the factors mentioned earlier are required for *Sporothrix* virulence and are also used for evasion of the host immune system. The knowledge of these virulence factors opens the possibility of understanding the differences in the virulence that exists between the *Sporothrix* species, as is the case for 9 out of 60 proteins differentially expressed in *S. brasiliensis* with respect to *S. schenckii*: aminopeptidase I, extracellular cell wall glucanase, Mn-superoxide dismutase, glyceraldehyde-3-phosphate dehydrogenase (GAPDH), heat shock 70-kDa protein 1/8, hydroxymethyl-glutaryl-coenzyme A (HMG-CoA) lyase, acetyl-CoA hydrolase, progesterone binding protein, and rhamnolipid biosynthesis 3-oxoacyl-(acyl-carrier-protein) reductase, where all of these are involved in the evasion of the host immune system [25]. Others exoantigens such as secreted proteins of 46, 60, 90, and 110 kDa are commonly secreted by *S. schenckii*, *S. brasiliensis*, and *S. globosa* [34].

## 4. Host Immune Response

### 4.1. Cellular Response

As has been mentioned already, the *S. schenckii* complex is composed of closely related fungi that cause sporotrichosis. These organisms are an interesting model to study the biochemical, genetic, molecular, and physiological basis of cell differentiation and morphogenesis. Differences in terms of the disease evolution have been reported in experimental models of sporotrichosis and in different *Sporothrix* clinical isolates [35,36]. Moreover, some studies have indicated that the immune response of the host determines the degree of invasion [37]. However, the mechanisms of evasion by the pathogen and control of the infection by the host are not yet clear.

**Figure 1:**
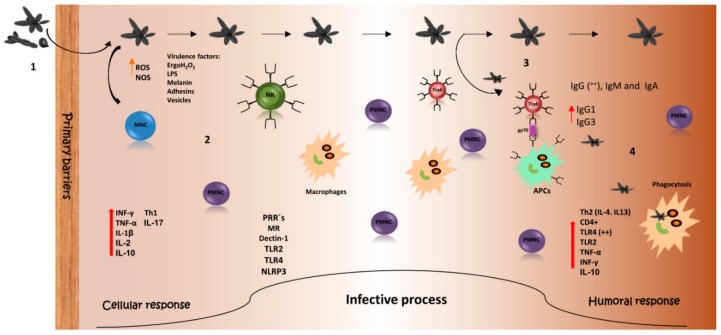
Host immune response against *Sporothrix* spp. (**1**) Infection with *Sporothrix* occurs by the traumatic inoculation of yeasts, conidia, or hyphae. Once the fungus crosses the primary barriers of the immune system, (**2**) components of the pathogen trigger the innate immune response including cells and molecules to attack the invader. (**3**) Several molecules of the fungus active T cells by being presented as antigens (i.e., peptides of Gp70). (**4**) Protective antibodies against the fungus are generated. All of this is for the clearance of the pathogen. MNC: Mononuclear cells. PMNC: Polymorphonuclear cells. NK: Natural Killer cells. Black arrows: infection sequence. Red arrows: increased production and participation during infection. Orange arrow: increased secretion during infection.

In order for the pathogen-host interaction to be carried out, the fungus needs to overcome the host first defense line, which involves the skin and mucous membranes, as shown in Figure 1. The innate immune response plays a key role in establishing an anti-*Sporothrix* protective response [38]. Phagocytosis by macrophages and neutrophils as well as the production of reactive oxygen species are mechanisms by which cells of *S. schenckii* are eliminated [39].

The cell wall (CW) of the pathogen plays a very important role because it contains the main points of contact with the host, which reacts with a mixed Th1/Th17 immune response able to confer resistance through the secretion of cytokines like IFN-γ, TNF-α, and IL-17A, that activate macrophages and neutrophils for fungal clearance. It has been reported that the CD4^+^ T cells provide host resistance by the secretion of the previously mentioned cytokines in other fungal infections such as candidiasis, aspergillosis, paracoccidioidomycosis, and coccidioidomycosis. Macrophages can be activated by IFN-β secretion during the Th1 response, considered the most important cytokine in sporotrichosis infection, given that it is able to confer protection by fungal killing. IL-17A is produced by Th17 cells and is involved in the repair and activation of epithelial barriers. Th17 cells are also crucial for the control of natural killer (NK) cells and antifungal defense [40,41,42,43].

The innate immune system allows for the rapid recognition of a broad spectrum of pathogens through the use of pattern recognition receptors (PRRs), and an effective immune response depends on macrophages that recognize pathogen-associated molecular patterns (PAMPs) [44]. Only a few characteristics of the PAMPs from the *Sporothrix* surface are known, for example, the cell wall lipids that are components with an important role in the pathogenesis of the fungus, which were found to inhibit the phagocytosis process increasing the stimulation of NO and TNF-α [45]. In other pathogens such as *P. brasiliensis*, the lipid components may play a role in the innate immunity against infection using Toll-dependent and independent mechanisms to control macrophage activation [46].

Other components such as ergosterol peroxide, cell-wall compounds (alkali-insoluble fraction and lipid extract), secreted fungal proteins, and exoantigens have the capacity to activate the innate immune system, produce the activation of reactive oxygen and nitrogen species (H_2_O_2_ and NO), and also activate the adaptive immune response in order to produce cytokines from the Th1 and Th2 profiles [47,48]. Ergosterol peroxide protects *S. schenckii* by helping to evade reactive oxygen species (ROS) during phagocytosis [37], a process mediated by macrophages; cells that in addition to secreting enzymes, complement components, coagulation factors, cytokines, ROS, and reactive nitrogen species (RNS) produce a powerful mediator of the inflammation and immune response, nitric oxide (NO). NO participates (in vitro) in *S. schenckii* destruction by macrophages and, contributes in vivo to the immunosuppression and balance of cytokines in early stages of sporotrichosis [49]. In addition, a role for NO has also been described in infection by *Histoplasma capsulatum* [50]. Activated bronchoalveolar and peritoneal macrophages kill yeast cells of *H. capsulatum* by a mechanism dependent on hydrogen peroxide and products of the nitric oxide synthase (NOS) pathway, whereas fungistasis depends only on products of the NOS pathway [50]. In *Cryptococcus neoformans*, the phenoloxidase enzyme system contributes to protect the fungus against nitrogen and oxygen-derived oxidative antimicrobial molecules produced by immune cells [51]. In *Paracoccidioides brasiliensis*, it was observed that NO is important for fungus clearance and contributes to the development of the immunosuppression observed during the course of the disease [52].

*S. schenckii* yeasts are capable of activating the complement, both of the classical and alternative pathways, the latter independently of the presence of antibodies [53,54]. Phagocytosis of conidia, but not yeasts, by macrophages indicates that the two morphologies are recognized by different receptors on the surface of the immune cells [55]. The participation of these receptors in primary immune cells has been analyzed, demonstrating that they are also relevant during the interaction of *Sporothrix* with the host. The interaction of *S. schenckii* and *S. brasiliensis* (conidia, germlings, and yeasts) with human peripheral blood monocyte cells (hPBMCs) is also dependent of TLR2, confirming that this receptor is relevant in both the murine and human response against these pathogens [56]. Animals lacking TLR2 produce lower levels of TNF-α, IL-1β, IL-2, and IL-10, addressing the importance of this receptor during the immune recognition of *S. schenckii* [55]. In addition, interaction experiments between hPBMCs with *Sporothrix* demonstrated a decrease of TLR4 participation when compared with the results in a murine model; most likely due to the nature of human and animal immune cells [56]. The participation of the receptors Dectin-1 and mannose receptor (MR) has also been reported [57].

Dectin-1 is a receptor described as relevant for the production of cytokines during the interaction of *S. schenckii sensu stricto*, and *S. brasiliensis* with hPBMCs, given that most of the β-1,3 glucans required to trigger Dectin-1 dependent signaling are already accessible on the surface of the cell [56]. The role of MR has been described as of greater importance for the phagocytosis of *S. schenckii sensu stricto*. It has a minor contribution to the stimulation of cytokines by yeasts and germlings, while the conidia morphotype stimulates the production of pro-inflammatory cytokines via the same receptor [55,56].

After *S. schenckii* recognition, NLRP3 (nucleotide-binding oligomerization domain-like receptor pyrin domain-containing 3), a receptor recently described, plays a critical role in combating this mycosis by mediating the Th response and linking the innate and adaptive immune responses [58].

Several antigenic peptides in the CW of *Sporothrix* have been described, and these are able to induce (in vitro) proliferation in T cells sensitized with *S. brasiliensis*. Among them are the ZR3 peptide, which is a sequence of an importin protein, the ZR4 peptide that comes from a hypothetical protein, and the ZR8, which is a peptide of the Gp70 glycoprotein [59]. The mentioned glycoprotein has been reported as the major adhesin in the CW and as the immunodominant molecule in sporotrichosis [60]. ZR3, ZR4, and ZR8 all induce high cell proliferation, but only ZR3 is able to induce the systemic production of high levels of IL-17A and IFN-γ. However, ZR8 is able to promote higher levels of IFN-γ, IL-17A, IFN-β, and IL-1β with a higher number of neutrophils in the lesions, and increases CD4^+^ T cells in the lymph nodes (with increased levels of IFN-β) and spleen with a higher number of neutrophils in the lesions, suggesting a Th1/Th17 immune response profile [59]. It is well known that the main mechanism for antibody protection is opsonization of the pathogen by the Fc gamma receptor (FcγR) [61]. This receptor mediates several macrophage functions such as the stimulation of cytokine secretion and activation of the respiratory burst. It was observed that when *Sporothrix* is phagocytosed in the presence of Ab against the Gp70 glycoprotein, the macrophages’ fungicidal ability is increased, with higher levels of the pro-inflammatory cytokines TNF-α, IL-1β, IL-6, and the anti-inflammatory cytokine IL-10 [61].

### 4.2. Humoral Response

In the past and for a long time, cell-mediated immunity was considered to be the fundamental mechanism of the host response against pathogenic fungi, and the protective role of humoral immunity was poorly understood. Recently, the participation of antibodies in host defense against fungal infection has been described and includes several mechanisms: agglutination of fungal cells, opsonization and enhancement of phagocytosis, inhibition of fungal cells attachment, complement activation and mediated lysis, neutralization of immunoregulatory molecules, Fc-mediated cytokine release, and antibody-dependent cellular cytotoxicity (ADCC) [62,63]. However, antibodies that do not confer protection or that even enhance the infection can be generated [64,65]. In addition, there are several factors that can affect the function of these antibodies such as isotype and concentration [64,66].

Despite the progress in the knowledge of the humoral response against pathogenic fungi, little is known about the antibody-mediated response against clinically relevant *Sporothrix* species [65,66], but it appears to participate in the induction of partially protective immunity and control of sporotrichosis in experimentally infected mice [64,65]. Cellular and humoral immune responses are important to achieve a protective state. The Th2 response is characterized by the presence of IL-4 and IL-13, which triggers T cell differentiation into the Th2 lineage with a decrease of the Th1 response and an increase of antibody production [7,32,67,68].

Due to its location, composition, and immunogenicity, the *Sporothrix* cell wall represents the major structure involved in the host–parasite interplay [65,68], and several cell wall proteins and secretory molecules expressed during infection have been reported as highly immunogenic and able to induce a protective response (Figure 1) [62,65].

A 2D-immunoblotting analysis was used for the identification of some antigenic proteins from the CW. With antibodies raised against the whole cell, two major immunogenic antigens were detected in the yeast morphology: the 60 and 70 kDa proteins. On the other hand, five major antigens were detected in the mycelial morphology of 48, 55, 66, 67, and 70 kDa. The 70 kDa protein, believed to be Gp70, was the main antigenic molecule present in both morphologies [69]. In addition, the antibodies against these antigenic components were accompanied with the production of cytokines characteristic of the Th1 and Th2 responses, high levels of IFN-γ and TNF-α, and an increased phagocytic index [63,66,70].

The antibody production that might be involved in the humoral response in experimental sporotrichosis in mice has been evaluated. The progress of the infection was monitored by organ culture, which revealed that fungal load increased in the first week post infection and decreased 14 days after infection. The higher fungal load at the initial phase of the infection suggests that resistance to *S. schenckii* does not depend on the capacity of the host to eliminate the pathogen, but instead is dependent on an acquired immunity developed with time. IgG antibodies against Gp70 or against soluble antigens were detected with a high concentration 14 days after infection and was maintained during the whole process. Isotyping of these antibodies showed the presence of IgG1 and IgG3, which could explain an important mechanism for antigen neutralization, since IgG1 and IgG3 are involved in opsonization and neutralization of *Sporothrix* yeasts by increasing macrophage efficiency, and therefore, decreasing colony forming units (CFUs) in organs [71]. These results might suggest that specific antibodies against this molecule participate in the control of the mycosis. With the production of the monoclonal antibody IgG1 against Gp70 (mAb P6E7) it was demonstrated that a protective response against experimental sporotrichosis could be established as shown in Figure 1, since mice passively immunized with the antibody (before, during, and after infection) had a significant reduction of fungal load in the spleen and liver, and it did not develop more severe forms of the disease [63,66,70]. The same response was observed in T-cell deficient mice (nude), given that the antibodies were able to control the infection in the absence of a T-cell response most likely through two mechanisms: an increase of the cell-mediated immunity and IFN-γ production; and/or inhibition of the yeast adhesion to the host tissue and extracellular matrix by the antibodies [32,70]. In addition, the fungicidal ability of macrophages is increased when the fungus is phagocytosed in the presence of mAb P6E7 or immune inactivated serum, with the observation that macrophages need at least 72 h to efficiently kill the yeast. This former mechanism was more evident in the case of mAb, indicating that the activation of macrophages by P6E7-opsonized yeast is late when compared with the immune serum-opsonized yeast, and therefore other factors in the immune serum, apart from anti-Gp70 antibodies, could participate in macrophage activation. Pro-inflammatory cytokines were analyzed, and the results showed a high production of TNF-α and IL-1β when the fungus was opsonized by the serum or mAb P6E7. With these results, it can be speculated that antibody-mediated phagocytosis may be essential for macrophage killing and production of TNF-α, and therefore the control of sporotrichosis [61].

The presence of IgG, IgM, and IgA antibody isotypes against *Sporothrix* mycelial exoantigens in serum from patients has also been described during sporotrichosis. These antibodies remained detectable during the treatment of the disease in most of the patients, but decreased over treatment time. It is important to mention that during treatment, the antibody levels differed depending on the clinical form; lower optical density (OD) values for IgM and IgA were detected with the fixed cutaneous and lymphocutaneous forms while no difference was observed in patients with disseminated cutaneous and extracutaneous forms [64]. This could be explained by the fungal load; in more severe cases, a higher fungal burden leads to continuous antigen presentation and therefore continuous antibody production. The presence of these antibodies during sporotrichosis has an important role in the pathogenesis of the disease since IgM has been shown to activate the complement by the classical pathway, and IgA participates in the case of mucosal involvement [64].

It has been previously reported that a specific immune response and resistance to infection can be developed after a previous infection or active immunization with *Sporothrix* cell wall proteins (CWP) or whole cells [62]. After immunization, antibodies against several fungal antigens can be detected, with Gp70 the best characterized [67]. Based on this, a vaccine using CWP was developed and tested in a mouse model. Immunization with aluminum hydroxide-adsorbed *S. schenckii* cell wall protein (AH + CWP100) induced specific IgG1 and IgG2a antibodies against CWP. IgG2 participates in several mechanisms including complement fixation and binding to Fcγ receptors to stimulate phagocytosis and ADCC in mice [62].

There is a lack of information about feline sporotrichosis and the antigenic molecules involved in the mycosis. Therefore, the antigens expressed, either by *S. schenckii* or *S. brasiliensis*, recognized by sera from cats naturally infected were detected. Cross-reactivity between the two species was found, recognizing the same antigens also identified by the human IgG response. The main molecules recognized by cats’ antibodies are Gp60 and Gp70 from *S. brasiliensis* and *S. schenckii*, respectively. No association between the severity and distribution of the lesions and the number or type of antigens recognized by the sera was found [72].

The antibody-mediated immunity was considered to be irrelevant in the host defense against *Sporothrix,* but experimental methods have been able to establish a fundamental role for humoral response in the prevention, control, and treatment of sporotrichosis.

## 5. Treatment

Sporotrichosis treatment depends on several factors including the clinical manifestation, the immune status of the host, and the causal species of *Sporothrix*. In antifungal susceptibility testing, the species from the *Sporothrix* complex respond differently to antifungals. Some reports of in vitro activity of antifungal agents against *Sporothrix* spp. have been evaluated, but the same effect has not always been observed in vivo, and only a few studies have correlated the therapeutic response and the susceptibility generated in vitro. It has been suggested that a combination of different antifungals could generate a favorable response [73]. In 2017, some of the antifungal agents were classified based on the susceptibility observed in species of the complex (Table 2). Potassium iodide and/or itraconazole (ITC) are the initial treatment for fixed cutaneous and lymphocutaneous sporotrichosis. It has been reported that terbinafine has activity against *S. schenckii sensu stricto* [74], and is considered as the second-line treatment for lymphocutaneous and cutaneous sporotrichosis. Amphotericin B is used in the disseminated, systemic, pulmonary, and osteoarticular forms [75]. A study of the activity in vitro against *S. brasiliensis* showed that recently isolated species between 2011 to 2012 had greater resistance to amphotericin B and ITC when compared with old isolates from 2004; the same was observed in 34% of recent isolates for posaconazole. Although terbinafine has been suggested as a potent antifungal against *S. brasiliensis*, more studies are needed [76].

Due to the differences in the antifungal susceptibility between species, the long period of time for the treatment, the high cost, and the collateral effects in the host, among other reasons, alternative therapies are required to treat infections by *Sporothrix* spp. Although ITC is the first choice to treat sporotrichosis [7], other drugs and the combination of these have been used to eradicate the fungus. Diphenyl diselenide (PhSe)_2_, alone or in combination with ITC, has demonstrated an efficient in vitro activity against *S. brasiliensis* [77], which is quite convenient given that (PhSe)_2_ shows less toxicity for mammal cells [78]. A case of canine sporotrichosis caused by *S. brasiliensis* was successfully cured using a combination of ITC plus β-1,3 glucan, the last one used as an immunomodulator [79]. Another compound used to treat sporotrichosis caused by *S. schenckii* ATCC 16345 was the ethanolic extract of *Vismia guianensis*, a tropical plant. The combination of the extracts plus ITC reduced the fungal load in the spleen of male Balb/c mice [80].

Regarding other treatment options, an in vitro assay based on photodynamic inactivation that produces ROS has been proven to be effective because it was demonstrated that it provokes a growth inhibition of the *Sporothrix* complex members, as was seen in conidia irradiated with laser light (energy dose of 28 J/cm^2^) in combination with different doses of methylene blue (0.5, 1.0, and 2.0 μg/mL). The species evaluated were *S. schenckii sensu stricto*, *S. albicans*, *S. brasiliensis*, *S. globosa*, and *S. mexicana*. This offers an alternative treatment to sporotrichosis, especially when caused by resistant-strains to antifungals. There is a report of a successful clinical case of a 65-year-old patient suffering recalcitrant cutaneous sporotrichosis, treated by the application of phototherapy (37 J/cm^2^ using the Aktilite lamp) with the administration of 1% methylene blue in the lesion, and in combination with low and intermittent doses of ITC, that allowed the clinical cure [81]. However, in vivo studies are required to demonstrate the effectiveness of this treatment [82].

As mentioned before, melanin, an important cell wall component for *Sporothrix* virulence, is another target to eliminate the fungus since the inhibition of the synthesis pathways of this component by tricyclazole increases the susceptibility of *S. brasiliensis* and *S. schenckii* against terbinafine; showing an alternative for the design of new antifungals that interfere in the synthesis of melanin, which can be applied as a combination therapy with other drugs [83]. In addition, *S. schenckii*, *S. brasiliensis*, *S. globosa*, and *S. mexicana* have the ability to produce a strong in vitro biofilm, which has sensitivity to amphotericin B and caspofungin [84].

There are studies focused on the generation of vaccines as antifungal therapies for sporotrichosis. In recent years, the participation of proteins present in the *Sporothrix* cell wall able to generate an immune response by the host that could be an alternative to obtain an immunoprotective effect has been evaluated. As mention previously, the monoclonal antibody against cell wall Gp70 glycoprotein has been used as a therapeutic vaccine in mice infected with highly virulent strains of *S. schenckii* and *S. brasiliensis*, obtaining a decrease in the fungal burden on the analyzed organs [63].

Recently, the immunoprotective effect of a recombinant phage with a peptide epitope (KPVQHALLTPLGLDR) of the Gp70 glycoprotein was demonstrated in mice infected with *S. globosa*, inducing a favorable cellular and humoral response for the control of the infection [85]. Finally, regarding the same alternative, the synthesized peptide from Gp70 glycoprotein (LKFLALASVISATSA) called ZR8 as mentioned previously, could be a candidate for vaccines against subcutaneous sporotrichosis. The peptide promotes increased CD4^+^ T cells and higher levels of cytokines (IFN-γ, IL-17A, and IL-1β) in mice infected with the yeast cells of *S. brasiliensis*. These recent advances are a new strategy for a possible vaccine for the treatment of sporotrichosis [59].

## 6. Conclusions

Over the last few years, significant progress has been made on the knowledge and understanding of the host immune interaction with the pathogenic species of *Sporothrix*. Much of the immune response during sporotrichosis is still undiscovered, but many virulence factors and PAMPs of the fungus are already known, information that has helped us to control the infection thus far. However, recent reports of hypervirulent strains prove the emergent importance of this fungus. The study of several immunogenic antigens, molecules, and the mechanism of the host immune cells can provide further development of new diagnostic tools, treatment protocols, and prevention strategies.

## Figures and Tables

**Table 1:** Several molecular studies for *Sporothrix* species identification.

Gene	Technique	Result
*CHS1*	PCR	Does not distinguish among species
18S rRNA	PCR	Does not distinguish among species
*ITS*	Sequence analysis	Distinguishes among species
*CAL*, *Bt2*, and *CHS*	Multilocus sequence analysis	Distinguishes among species
Calmodulin	Partial gene sequencing	Distinguishes among species
*T3B* primer	PCR fingerprinting	Distinguishes among species
Calmodulin digested with *Hha*I	PCR-RFLP	Does not distinguish between *S. mexicana* and *S. pallida*
*CAL* introns	PCR	Distinguishes among species
Calmodulin	PCR-based rolling circle amplification	Distinguishes among species

**Table 2:** Drugs evaluated in vitro for *Sporothrix* species.

Sporothrix Complex	Good *	Moderate *	Low *
*S. brasiliensis*	Itraconazole, posaconazole, terbinafine, and potassium iodide	Amphotericin B	Caspofungin, voriconazole, fluconazole and flucytosine
*S. schenckii*	Amphotericin B, posaconazole and terbinafine	Itraconazole	Voriconazole, fluconazole and echinocandins
*S. globosa*	Terbinafine	Fluconazole and voriconazole	Itraconazole
*S. mexicana*	Terbinafine, ketoconazole	Amphotericin B	Posaconazole

* Activity antifungal reported.
